# Smart optical cross dipole nanoantenna with multibeam pattern

**DOI:** 10.1038/s41598-021-84495-0

**Published:** 2021-03-03

**Authors:** Seyyed Mohammad Mehdi Moshiri, Najmeh Nozhat

**Affiliations:** Department of Electrical Engineering, Shiraz University of Technology, 7155713876 Shiraz, Iran

**Keywords:** Nanophotonics and plasmonics, Optical properties and devices

## Abstract

In this paper, an optical smart multibeam cross dipole nano-antenna has been proposed by combining the absorption characteristic of graphene and applying different arrangements of directors. By introducing a cross dipole nano-antenna with two V-shaped coupled elements, the maximum directivity of 8.79 dBi has been obtained for unidirectional radiation pattern. Also, by applying various arrangements of circular sectors as director, different types of radiation pattern such as bi- and quad-directional have been attained with directivities of 8.63 and 8.42 dBi, respectively, at the wavelength of 1550 nm. The maximum absorption power of graphene can be tuned by choosing an appropriate chemical potential. Therefore, the radiation beam of the proposed multibeam cross dipole nano-antenna has been controlled dynamically by applying a monolayer graphene. By choosing a suitable chemical potential of graphene for each arm of the suggested cross dipole nano-antenna without the director, the unidirectional radiation pattern shifts ± 13° at the wavelength of 1550 nm. Also, for the multibeam nano-antenna with different arrangements of directors, the bi- and quad-directional radiation patterns have been smartly modified to uni- and bi-directional ones with the directivities of 10.1 and 9.54 dBi, respectively. It is because of the graphene performance as an absorptive or transparent element for different chemical potentials. This feature helps us to create a multipath wireless link with the capability to control the accessibility of each receiver.

## Introduction

Nowadays, smart and optical multibeam nano-antennas have attracted the attention of many scientific researches considerably in the current science and engineering of nanophotonic technology^1–3^. Optical multibeam nano-antennas (OMBNAs) have capability to radiate multiple independent beams simultaneously from a single orifice, which leads to the creation of a multi-user wireless access link^4,5^. One contributed approach to design OMBNAs is smart control of the accessibility of each user or receiver that can be realized by utilizing the unique characteristics of graphene to manipulate the radiation beams from the transmitter^6–11^. The idea of optical multibeam nano-antennas has been extracted from the traditional microwave ones. In conventional microwave antennas, multibeam antennas have variety applications in radar and communication systems and cellular mobile. In contrast, OMBNAs cannot transmit the radiation pattern over a long distance because of the inherent loss of metal at optical frequencies^12,13^. OMBNAs are appropriate to decrease the fabrication complexity of integrated on-chip circuits due to making multipath wireless links between different elements of photonic integrated circuits (PICs)^14,15^. As a result, the most important application of OMBNAs is utilizing them as an intra or inter on-chip wireless link to transmit optical data from one device to other devices placed at different chips or layers^16,17^.

Up to now, numerous types of nano-antennas have been proposed such as circular hybrid plasmonic^18^, Yagi-Uda^19^, dipole-loop^20^, graphene patch^21^, and phased array^22^ antennas. However, to the best of our knowledge, a few efforts have been done to investigate optical nano-antennas with multibeam radiation pattern. One approach to have an OMBNA is considering a long rectangular coupled dipole nano-antenna, which supports higher-order resonances^12^. Although, bi- and quad-directional patterns can be attained based on this method, the length of antenna should be increased. By increasing the length of rectangular coupled dipole nano-antenna, total emission rates of higher-order resonances are decreased, which leads to reducing the antenna efficiency and creation of wider radiation pattern in some cases^12^. It is because of reduction of SPPs amplitude at the interface of metal and dielectric. The other method to propose an OMBNA is enhancing the second harmonic (SH) radiation of the nano-antenna, which is a critical challenge in nonlinear optics^23^. In this regard, by changing the material of substrate such as epsilon-near-zero, gold and AlGaAs, the shape of radiation pattern at the SH wavelength has been modified by applying a high input intensity. By choosing an appropriate input intensity, not only the permittivities of the materials used in^23^ have been changed, but also the target wavelength varies from 1550 to 750 nm. Consequently, by changing the intensity of illuminated plane wave, the permittivity of chosen substrate varies, which has impact on the radiation pattern. However, this method has some disadvantages such as changing the radiated wavelength and having limitation to choose materials based on their dielectric strength^23^ and control of input intensity to have the SH feature. By mentioned static approaches to design OMBNAs, the form of radiation pattern changes, but this change is stable and cannot be controlled or modified intelligently.

The necessity of directivity enhancement of OMBNAs, which plays a critical role in design of point-to-point wireless link, leads to utilizing the ideas of array configurations and directors^24,25^. Moreover, controlling the access of each receiver to radiated data is a major requirement of multi-user nano-systems, which boosts the reliability and information security^26^. In this regard, design of smart OMBNA, which works as a controllable switch, is necessary to make a decision which beam must be on and which one should be off.

The power absorption of graphene at optical frequencies can be tuned by its chemical potential, which can be controlled via manipulating chemical and electrostatic doping^27,28^. Consequently, to propose a smart nano-antenna, graphene is an efficient choice. Different graphene-based nano-antennas have been studied, but none of them have used the chemical potential of graphene to control the radiation pattern. In previous works, graphene have been only used for beam formation in the terahertz band^26,27,29^.

In this paper, for the first time, the idea of cross dipole has been proposed to develop the directivity of conventional dipole nano-antenna from 2.46 to 8.79 dBi at the wavelength of 1550 nm. It has been shown that by applying the directors with different configurations at the end of each arm of cross dipole nano-antenna, the type of radiation pattern has been controlled statically. Each director consists of two or three circular sectors, which leads to the creation of bi- and quad-directional patterns. Indeed, by applying the directors, each arm radiates separately or the radiation power from each arm couples to each other and builds a uniform radiation pattern at the desired direction. Changing the arrangement of directors is a static one to control the beam radiation. However, in OMBNAs dynamic control of radiation pattern is essential to tune the accessibility of the multipath wireless links. As a result, by choosing the best value of chemical potential of a 0.5 nm monolayer graphene at 1550 nm, which has coated the proposed cross dipole nano-antenna, the radiation pattern has been controlled successfully. In this regard, not only the radiation direction of unidirectional pattern of cross dipole nano-antenna without the director experiences ± 13° shifts, but also the bi- and quad-directional patterns of cross dipole nano-antenna with the director have been modified to uni- and bi-directional patterns, respectively, at 1550 nm. To attain the aim of controllability of radiation pattern of suggested OMBNA, each arm must have a separate electrostatic doping to tune the chemical potential separately. Consequently, by considering this approach, we have finally succeeded to design a smart cross dipole nano-antenna to control the radiation pattern to manage the accessibility of multipath wireless links.

## Characteristics and performance of cross dipole nano-antenna

**Figure 1:**
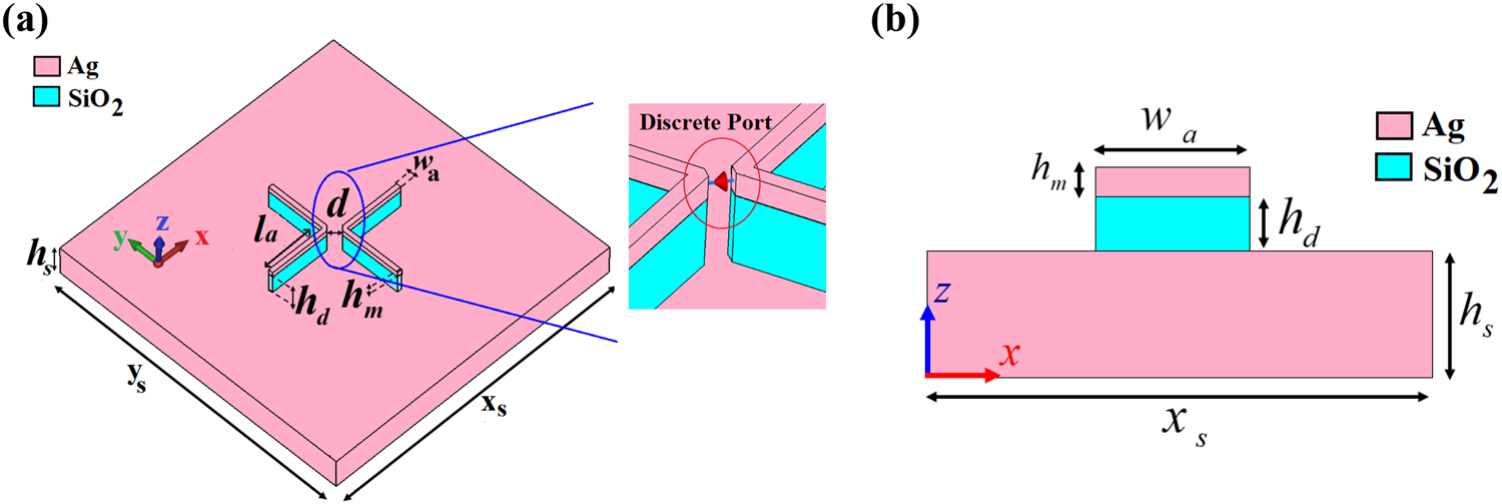
(**a**) The 3D perspective and (**b**) cross-section views of cross dipole nano-antenna without the director with 3D zoom in to show the discrete source as an illumination port. The thickness of substrate and foot-print are *h*_*s*_ = 220 nm and x_s_ × y_s_ = 3500 × 3500 nm^2^, respectively.

Figure 1 shows the three-dimensional (3D) and cross-section views of the proposed V-shaped cross dipole nano-antenna. It is composed of a silica (SiO_2_) layer grown between the silver (Ag) cap and metal substrate. The thicknesses of Ag cap and SiO_2_ layers are *h*_*m*_ = 35 nm and *h*_*d*_ = 120 nm, respectively. The length and width of each arm are *l*_*a*_ = 395 nm and *w*_*a*_ = 30 nm, respectively. Each V-shaped arm is placed at the distance of *d* = 80 nm from each other. The permittivities of Ag and SiO_2_ layers are extracted from the experimental data of Johnson-Christy and Palik, respectively^30,31^. The proposed nano-antenna is illuminated by a discrete port that can be considered as a quantum dot source to stimulate the nano-antenna^32^. This is because of the importance of quantum dot illumination as a secure communication and quantum computation protocols in the low-loss wavelength region around 1550 nm (193.5 THz) for the development of device-based quantum network infrastructure^33^. The discrete port is defined by a starting point and an end point. These two points will be connected by a perfectly conducting wire (visualized by a thick blue line) and the respective port source (indicated by a red cone) in the center of this wire, as shown in Fig. 1a. The starting and end points are connected to the Ag layer, which is parallel to the Ag substrate. Also, the wire between the starting point and the end point of the discrete element must be located along mesh edges. The radiation pattern of the discrete port without the nano-antenna structure is like a monochromatic, horizontal half-wave dipolar emitter dipolar emitter with the omnidirectional radiation pattern, 50 Ω internal impedance and 1 W input power. The electrical length of the port should be less than *λ*/10, otherwise the simulation results will differ greatly.

In comparison to some previous published nano-antennas, instead of a gold cap layer, consideration of silver is one of the superiorities of the proposed cross dipole nano-antenna. It is because of smaller total damping rate (Γ = 0.02 eV), lower loss, low fabrication cost and much better surface plasmon polaritons (SPPs) stimulation at telecommunication wavelengths^34,35^.

To investigate the performance of the suggested V-shaped cross dipole nano-antenna, the wavelength of 1550 nm has been chosen because of its importance in optical communication wireless links for intra and inter on-chip devices due to the low attenuation and eye safety^34^.

**Figure 2:**
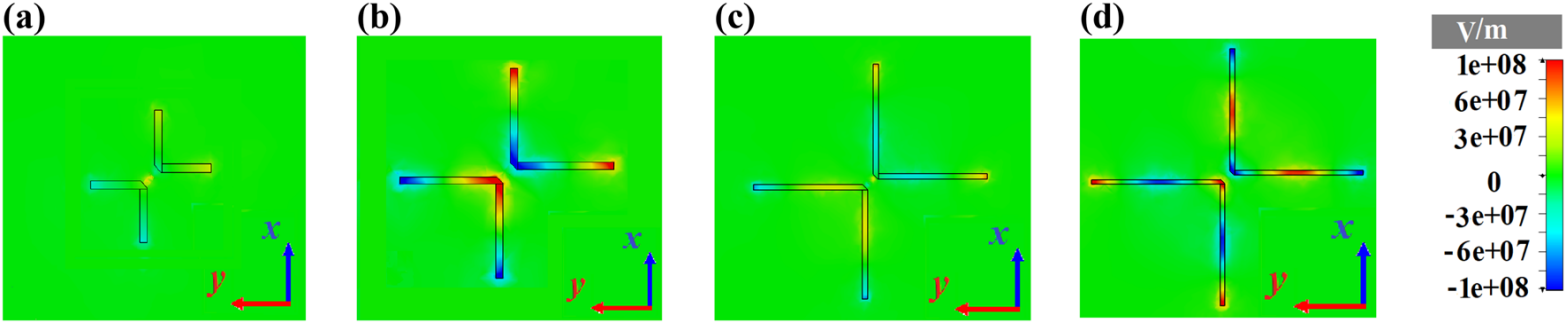
Plasmonic behavior of cross dipole nano-antenna without the director at 1550 nm as a function of *l*_*a*_. Distributions of the z-component of electric field in the xy plane of the nano-antenna for (**a**) ${{\lambda_{g} } \mathord{\left/ {\vphantom {{\lambda_{g} } 4}} \right. \kern-\nulldelimiterspace} 4}$, (**b**) ${{\lambda_{g} } \mathord{\left/ {\vphantom {{\lambda_{g} } 2}} \right. \kern-\nulldelimiterspace} 2}$, (**c**) ${{3\lambda_{g} } \mathord{\left/ {\vphantom {{3\lambda_{g} } 4}} \right. \kern-\nulldelimiterspace} 4}$ and (**d**) $\lambda_{g}$.

Because of the Ag-SiO_2_ interface in the proposed cross dipole nano-antenna, the SPPs should be excited. To verify this prediction and confirm the plasmonic behavior of the nano-antenna, the z-component of electric field for different electrical lengths at 1550 nm is depicted in Fig. 2. It can be seen that by choosing the length of cross dipole nano-antenna as $l_{a} = \frac{{\lambda_{g} }}{2} = \frac{{\lambda_{0} }}{{2n^{\prime}_{eff} }} = 403\,\,{\text{nm}}$, where $\lambda_{0}$ is the radiated wavelength of nano-antenna, the SPPs have been stimulated strongly with respect to other lengths of cross dipole nano-antenna. The effective refractive index of cross dipole nano-antenna at 1550 nm is $n_{eff} = n^{\prime}_{eff} + jn^{\prime\prime}_{eff} = 1.92 + j0.012$, which is obtained by mode solution analysis based on the finite difference time domain (FDTD) simulation. The considered length is a bit less than the predicted value by study the field distribution. This difference is related to the effect of field fringing in the case of 3D numerical simulation to obtain the electric field, which is more than the field fringing to calculate the two dimensional (2D) numerical result by the mode solver. The Lumerical software has been used to compute the effective refractive index at the cross-sections of guiding structures for a given frequency. Hence, we have designed the cross-section of our proposed cross dipole nano-antenna, shown in Fig. 1b, to compute the effective refractive index at the frequency of 193.5 THz. In order to obtain the precise results, the perfectly matched layer (PML) boundary condition with 20 layers has been considered for the mode solution analysis.

According to Fig. 2, it is obvious that two anti-parallel currents are flowing along each arm with odd symmetry. The propagated SPPs at the end of each arm can emit effectively to free space to create a uniform radiation pattern.

**Figure 3:**
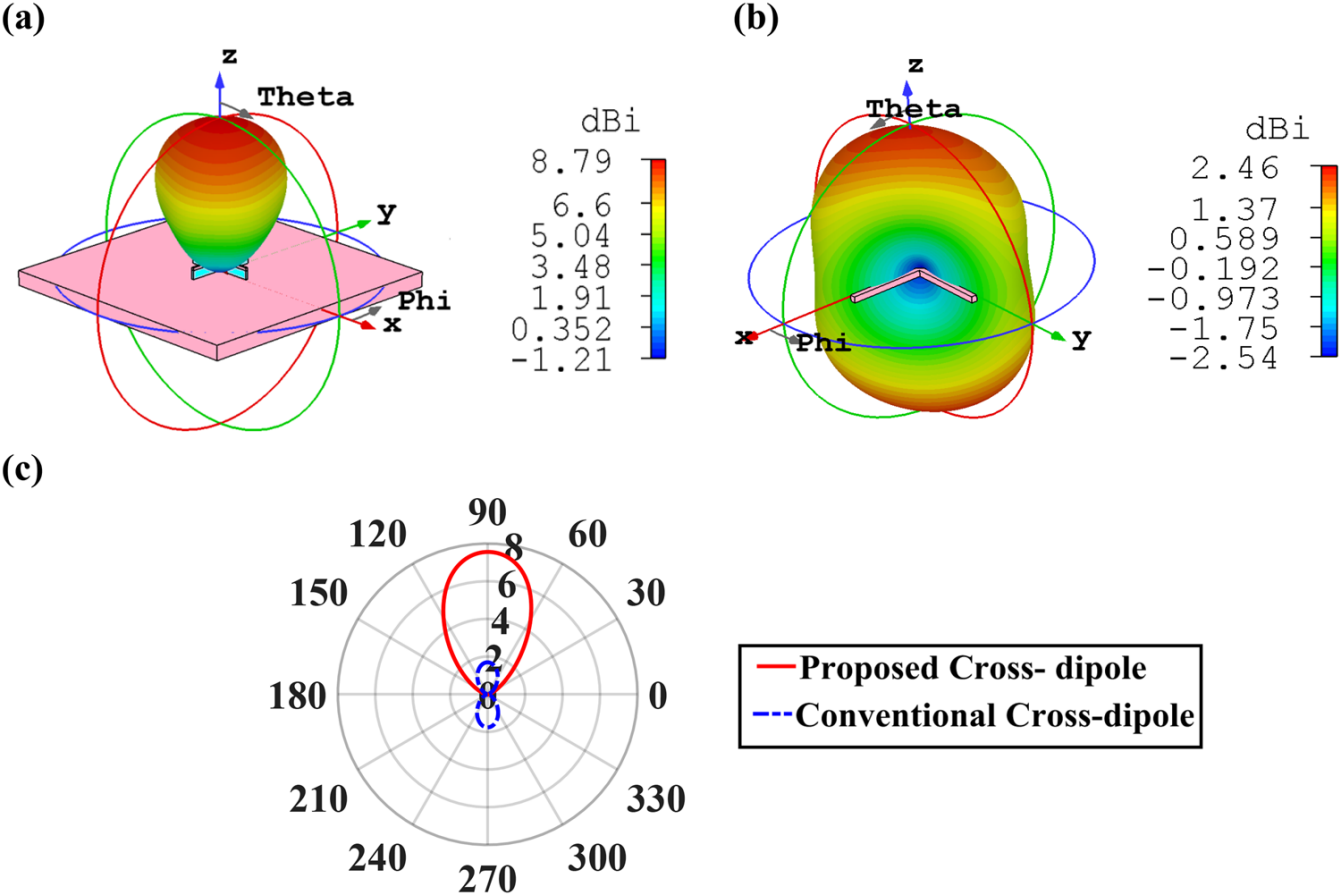
3D directivity radiation patterns of (**a**) the proposed cross dipole nano-antenna of Fig. 1 and (**b**) conventional metal cross dipole nano-antenna. (**c**) 2D polar plot of directivity radiation pattern of the proposed and convention cross dipole nano-antennas.

3D directivity radiation pattern of the introduced cross dipole nano-antenna is illustrated in Fig. 3a, which the obtained directivity is 8.79 dBi. To show the advantage of the proposed nano-antenna and explain why the use of metal substrate and growing the metal cap on the silica layer play a crucial role, it is essential to compare it with the conventional metal cross dipole nano-antenna. Figure 3b depicts that the conventional metal cross dipole nano-antenna has a bidirectional pattern with the maximum directivity of 2.46 dBi. Obviously, by utilizing the idea of metal substrate, the radiation pattern from the substrate has been omitted, which it could damage the other integrated devices on the platform. Besides, by introducing this structure, the obtained directivity of unidirectional pattern is enhanced more than 3.57 times compared with the conventional cross dipole nano-antenna with the directivity of 2.46 dBi.

As shown in Fig. 3c, the attained unidirectional pattern has the main lobe direction, 3 dB angular width (half-power beamwidth of the nano-antenna radiation pattern) of E-plane and side lobe level of 1°, 68.1° and − 24.4 dB, respectively, which verifies the better performance of the cross dipole nano-antenna in comparison to the conventional one.

Analogous to microwave antennas, applying directors helps us to manipulate the shape of radiation pattern. Also, in contrast to conventional horizontal rectangular director in microwave antennas, we have used circular director. Unlike the microwave antenna, using directors does not have major effect on the enhancement of directivity. Basically, it is because of the current penetration inside the metal and metallic loss at near-infrared frequencies^36^. However, its impact on changing the shape of radiation pattern is significant.

**Figure 4:**
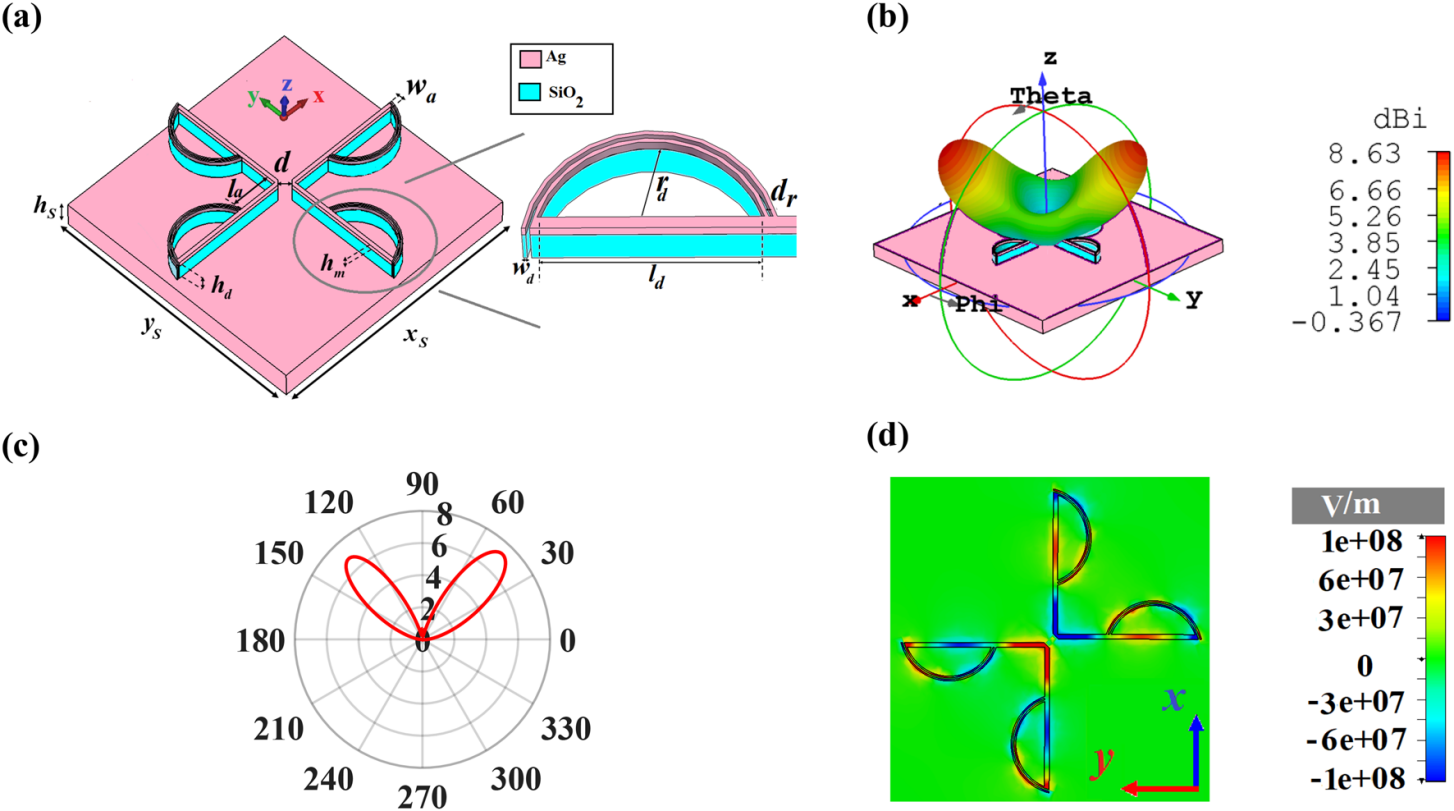
(**a**) 3D perspective view of bidirectional cross dipole nano-antenna with circular directors. (**b**) 3D and (**c**) 2D polar plot of directivity radiation patterns and (**d**) the z-component of electric field distribution.

As depicted in Fig. 4a, the cross dipole nano-antenna with the director consists of two circular sectors as radiators at the end of each arm of nano-antenna with the width of *w*_*d*_ = 10 nm. Also, the distance between two sectors is *d*_*r*_ = 10 nm. Each director is composed of a metal layer on top of the SiO_2_ layer. To obtain the optimized directivity, the lengths of cross dipole and directors are considered as *l*_*a*_ = 303 nm and *l*_*d*_ = 554 nm, respectively. Also, *r*_*d*_ = 205 nm, x_s_ × y_s_ = 3500 × 3500 nm^2^ and the other structural parameters are the same as Fig. 1.

3D radiation pattern of the cross dipole nano-antenna with the director in Fig. 4b reveals that by adding four circular directors, the unidirectional radiation pattern has been modified to bidirectional one with the maximum directivity of 8.63 dBi. Also, based on 2D polar plot of bidirectional nano-antenna, which is illustrated in Fig. 4c, the main lobe direction and 3 dB angular width are ± 44° and 35.3°, respectively, at the wavelength of 1550 nm.

To better understand the performance of adding directors to change the radiation pattern, the z-component of electric field distribution of the proposed nano-antenna with circular sectors is studied in Fig. 4d. It is clear that the excited SPPs along the V-shaped arms lead to the flow of anti-parallel currents in opposite directions. Therefore, the destructive effect of anti-parallel currents prevents the radiation of V-shaped arms and their emissions override. This effect happens when the electrical length of each V-shaped arm is $l_{a} = \frac{{\lambda_{0} }}{{2n^{\prime}_{eff} }}$. In contrast, the stimulated currents of two adjacent circular sectors are in-phase that makes the excited SPPs of each director to emit to free space with similar behavior. Consequently, the emitted lightwave from each adjacent director couples together constructively and creates a separate uniform radiation pattern, which leads to having a bidirectional nano-antenna. It is noteworthy that besides the in-phase currents, one important attribute for coupling of the emitted radiation patterns is the arrangement of circular directors. The coupled transmission line between the beginning and the end of circular sectors, denoted by *l*_*d*_, fortifies the power of emitted SPPs to free space. As a result, through the strengthening of the radiated power of each adjacent director, they can overcome the free space path loss and couple to each other, easily.

**Figure 5:**
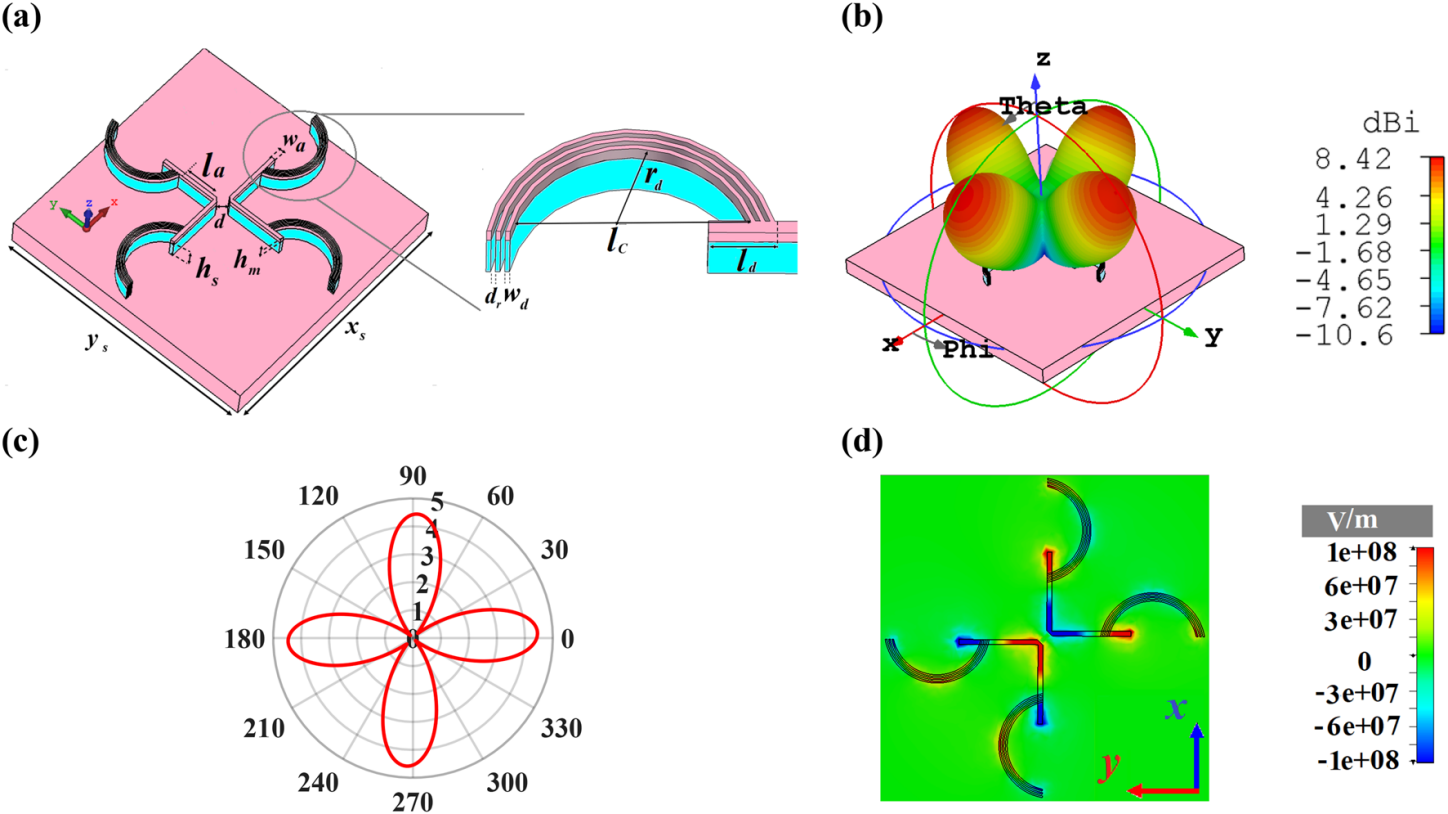
(**a**) 3D perspective view of quad-directional cross dipole nano-antenna with the director. (**b**) 3D and (**c**) 2D polar plot of directivity radiation patterns and (**d**) the z-component of electric field distribution. *r*_*d*_ = 185 nm, *l*_*d*_ = 177 nm, *l*_*a*_ = 303 nm, *l*_*c*_ = 512 nm and x_s_ × y_s_ = 3500 × 3500 nm^2^. The other structural parameters are the same as Fig. 1.

As shown in Fig. 5a, by exerting three circular sectors as director at the end of each arm of cross dipole nano-antenna with different arrangement, a quad-directional nano-antenna is designed. The attained results based on the 3D and 2D directivity radiation patterns of Figs. 5b,c disclose that the antenna can effectively emit to four directions to create an OMBNAs with the maximum directivity of 8.42 dBi. The z-component of electric field distribution of the cross dipole nano-antenna with three circular sectors is demonstrated in Fig. 5d. It is obvious that by omitting the plasmonic transmission line between the beginning and the end of directors, the emitted SPPs cannot overcome the free space path length. For this reason, they cannot couple to each other to form a uniform radiation pattern in each side. Therefore, each director radiates separately leading to a quad-directional nano-antenna.

**Figure 6:**
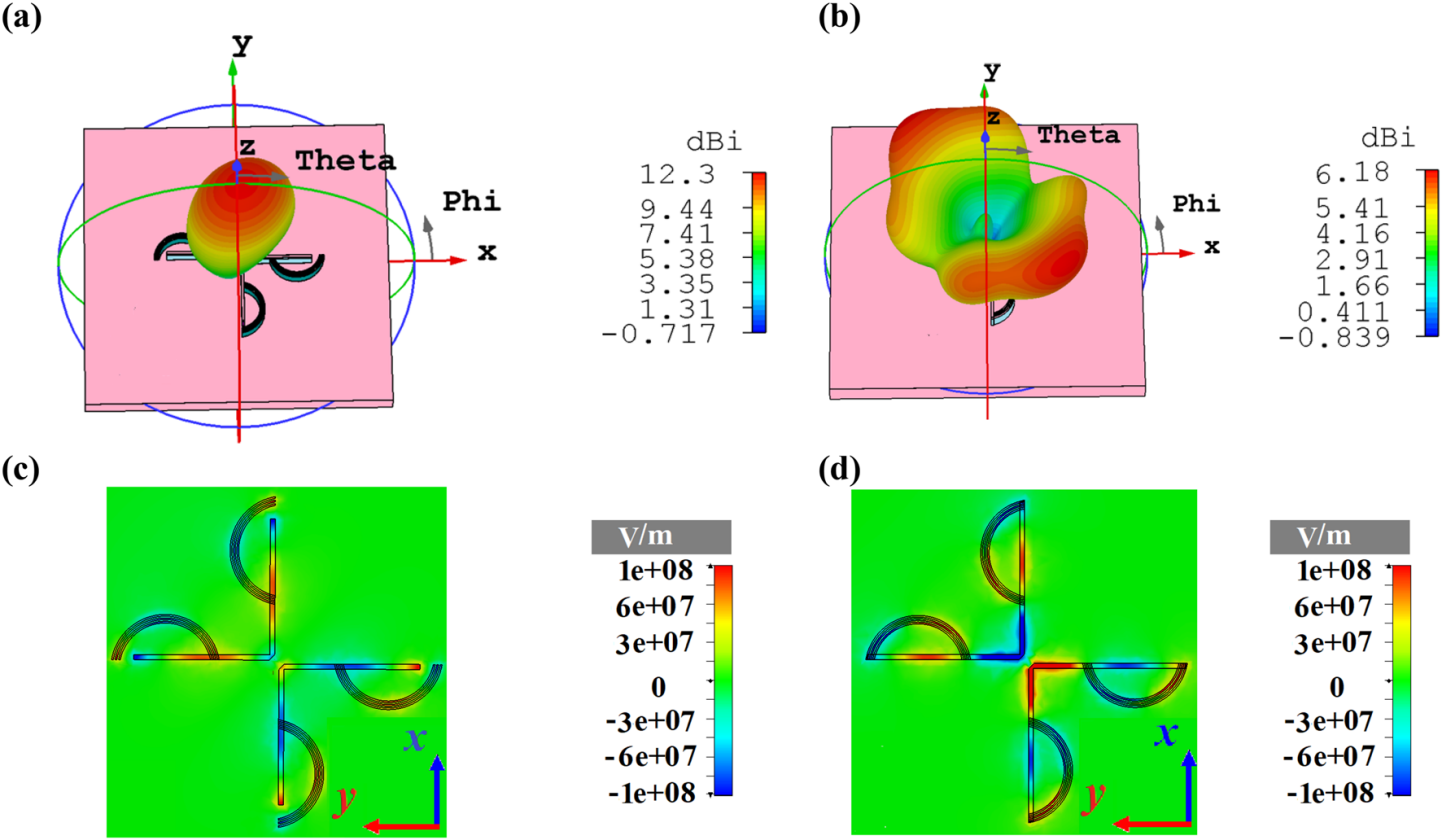
3D radiation patterns of cross dipole nano-antenna with three circular sectors at 1550 nm for (**a**) *l*_*d*_ = 517 nm, (**b**) *l*_*d*_ = 617 nm. The z-component of electric field distributions at 1550 nm for (**c**) *l*_*d*_ = 517 nm and (**d**) *l*_*d*_ = 617 nm.

One of the most important structural parameters for controlling the type of radiation pattern statically is *l*_*d*_*.* The 3D radiation patterns of the quad-directional cross dipole nano-antenna for two values of *l*_*d*_ = 517 nm and *l*_*d*_ = 617 nm at the wavelength of 1550 nm are illustrated in Fig. 6. According to Fig. 6a, when *l*_*d*_ = 517 nm, a unidirectional radiation pattern with the directivity of 12.3 dBi is obtained. Also, for *l*_*d*_ = 617 nm, the quad-directional radiation pattern of the cross dipole nano-antenna changes to a bidirectional one with the directivity of 6.18 dBi, as shown in Fig. 6b. However, in this case, in order to enhance the directivity from 6.18 to 8.63 dBi (Fig. 4b), the idea of two circular sectors can be used.

The gap between the coupled transmission line and the end of director plays a major role in the formation of radiation pattern. The gap acts as a capacitor, which provides the phase difference^16^ to control the coupling between the emitted SPPs from the transmission line and directors. Therefore, it has an important effect on the coupling power of two adjacent directors. Based on the obtained result of Fig. 6c, as excepted, each arm radiates separately, but the provided phase difference based on the virtual capacitor can steer the radiation beam to couple to each other to have a unidirectional radiation pattern. Furthermore, by increasing *l*_*d*_, the value of virtual capacitor is increased, which leads to a change in the phase of excited currents and so the beam steering happens. As the provided phase is not sufficient to steer the radiated beams to couple to each other effectively, a destructive coupling happens. Therefore, as shown in Fig. 6d, the directivity decreases from 12.3 to 6.18 dBi and a bidirectional radiation pattern is formed. As a result, we can tune the type of radiation pattern statically. However, in order to enhance the superiority of the proposed cross dipole nano-antenna, a dynamic approach will be discussed in the following.

## Characteristic of a monolayer graphene to control the absorption power

To demonstrate the hypothesis of smart OMBNAs, graphene is a good candidate because of its significant characteristics as a gate-variable optical conductivity that provides controllable devices such as modulator, nano-antenna and so on^37^. The complex optical conductivity of graphene $\left( {\sigma_{g} (\omega ,\mu ,\Gamma ,T)} \right)$, which is related to the chemical potential $\left( \mu \right)$, charge particle scattering rate $\left( \Gamma \right)$, angular frequency $\left( \omega \right)$, and temperature (*T*), can be defined by contributions of both inter-band and intra-band transitions from the Kubo formula^27^:

1 $$\sigma_{g} = \sigma_{{{\text{intra}}}} + \sigma_{{{\text{inter}}}}$$

where

2 $$\sigma_{{{\text{intra}}}} (\omega ,\mu ,\Gamma ,T) = - j\frac{{e^{2} K_{B} T}}{{\pi \hbar^{2} \left( {\omega - j\Gamma } \right)}}\left( {\frac{\mu }{{K_{B} T}} + 2\ln \left( {e^{{ - \frac{\mu }{{K_{B} T}}}} + 1} \right)} \right)$$

3 $$\sigma_{{{\text{inter}}}} (\omega ,\mu ,\Gamma ,T) = \frac{{ - je^{2} }}{{4\pi \hbar^{2} }}\ln \left( {\frac{{2\left| \mu \right| - \left( {\omega - j\Gamma } \right)\hbar }}{{2\left| \mu \right| + \left( {\omega - j\Gamma } \right)\hbar }}} \right)$$

in which reduced Plank constant, electron charge, and Boltzmann constant are shown by $\hbar$, *e*, and *K*_*B*_, respectively. Also, the complex dielectric function of graphene with the thickness of $\Delta$ = 0.5 nm and free space permittivity of ε_0_ is given by^37^:

4 $$\varepsilon_{g} \left( {\omega ,\mu ,\Gamma ,T,\Delta } \right) = 1 + \frac{{j\sigma_{g} }}{{\omega \varepsilon_{0} \Delta }}$$

The room temperature and charge particle scattering rate are considered to be *T* = 300 K and $\Gamma = 5\,\,\left( {{\text{ps}}} \right)^{ - 1}$, respectively.

**Figure 7:**
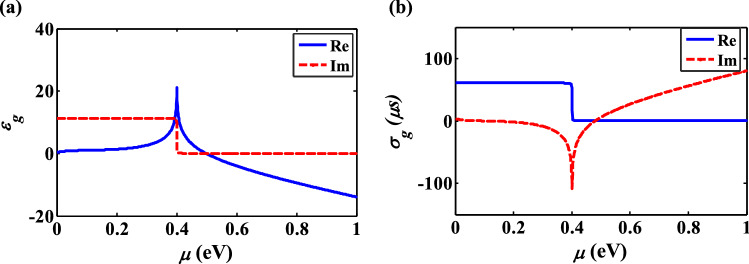
(**a**) Dielectric permittivity and (**b**) conductivity of graphene as a function of chemical potential at 1550 nm.

The dielectric permittivity and conductivity of graphene as a function of chemical potential at 1550 nm are shown in Fig. 7. It can be inferred that the transition chemical potential of graphene at 1550 nm is $\mu = 0.51\,\,{\text{eV}}$ that $\left| {\varepsilon_{g} \left( {\mu = 0.51\,\,{\text{eV}}} \right)} \right| = \left| { - 0.048 + j0.323} \right|$ is nearly 35 times less than $\left| {\varepsilon_{g} \left( {\mu = 0\,\,{\text{eV}}} \right)} \right| = \left| {0.3553 + j11.31} \right|$. This phenomenon realizes as epsilon-near-zero effect of graphene, which can be tuned by electrical gating^38,39^. As a result, study the effect of transition chemical potential of graphene on the absorbed power is a necessity that will be investigated in the following.

**Figure 8:**
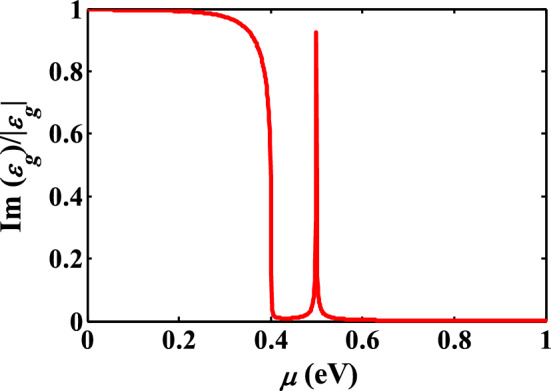
The ratio of ${\text{Im}} \left( {\varepsilon_{g} } \right)$ and $\left| {\varepsilon_{g} } \right|$ as a function of chemical potential at of 1550 nm.

The variation of dielectric constant by chemical potential can be used to design a controllable plasmonic device, which switches between the low- and high-power absorption, according to $P_{g} = 0.5{\text{Re}} \left( {\sigma_{g} } \right)E^{2} \propto 0.5E\left( {{{{\text{Im}} \left( {\varepsilon_{g} } \right)} \mathord{\left/ {\vphantom {{{\text{Im}} \left( {\varepsilon_{g} } \right)} {\left| {\varepsilon_{g} } \right|}}} \right. \kern-\nulldelimiterspace} {\left| {\varepsilon_{g} } \right|}}} \right)$ formula^38^. To verify the results of Fig. 7 and show the importance of epsilon-near-zero effect of graphene on the absorptive performance of the optical devices, it is essential to study the absorbed power by the graphene as a function of its chemical potential. To show in which chemical potential graphene becomes absorptive at 1550 nm, the value of ${{{\text{Im}} \left( {\varepsilon_{g} } \right)} \mathord{\left/ {\vphantom {{{\text{Im}} \left( {\varepsilon_{g} } \right)} {\left| {\varepsilon_{g} } \right|}}} \right. \kern-\nulldelimiterspace} {\left| {\varepsilon_{g} } \right|}}$ as a function of chemical potential is depicted in Fig. 8. Basically, if $\mu < \frac{\hbar \omega }{2}$, the inter-band term dominates and the power absorption of graphene reaches its maximum value.

When $\mu = 0.51\,\,{\text{eV}}$, the absorbed power reaches its maximum, which confirms the obtained results of Fig. 7. Therefore, in our case, at $\mu = 0\,\,{\text{eV}}$ and $\mu = 0.51\,\,{\text{eV}}$, graphene acts as a transparent and absorptive media, respectively. In the next section, we have used this unique feature of graphene to design a smart nano-antenna to control the direction of radiation beam.

## Performance of the intelligent multibeam cross dipole nano-antenna

**Figure 9:**
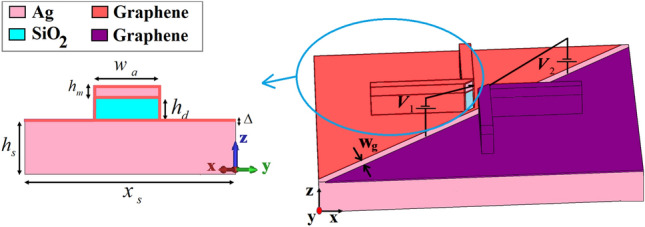
3D schematic view of smart unidirectional cross dipole nano-antenna.

As mentioned before, by changing the electrical gating, the chemical potential and plasma frequency of graphene can be controlled to tune the absorbed power. However, the effect of dielectric constant variation of graphene is not very obvious when it is placed on top of the dielectric layer. Consequently, not only the graphene layer is placed on top of the metal substrate and between the metal cap and dielectric layer, but also the proposed cross dipole nano-antenna is covered with the monolayer graphene, as illustrated in Fig. 9, to enhance its effect. Besides, in order to control the radiation of illumination source, the middle of metal substrate with the width of *w*_*g*_ = 59 nm is not covered with graphene. To control the main lobe direction of radiation pattern of unidirectional cross dipole nano-antenna, each side of nano-antenna is controlled with different chemical potentials.

**Figure 10:**
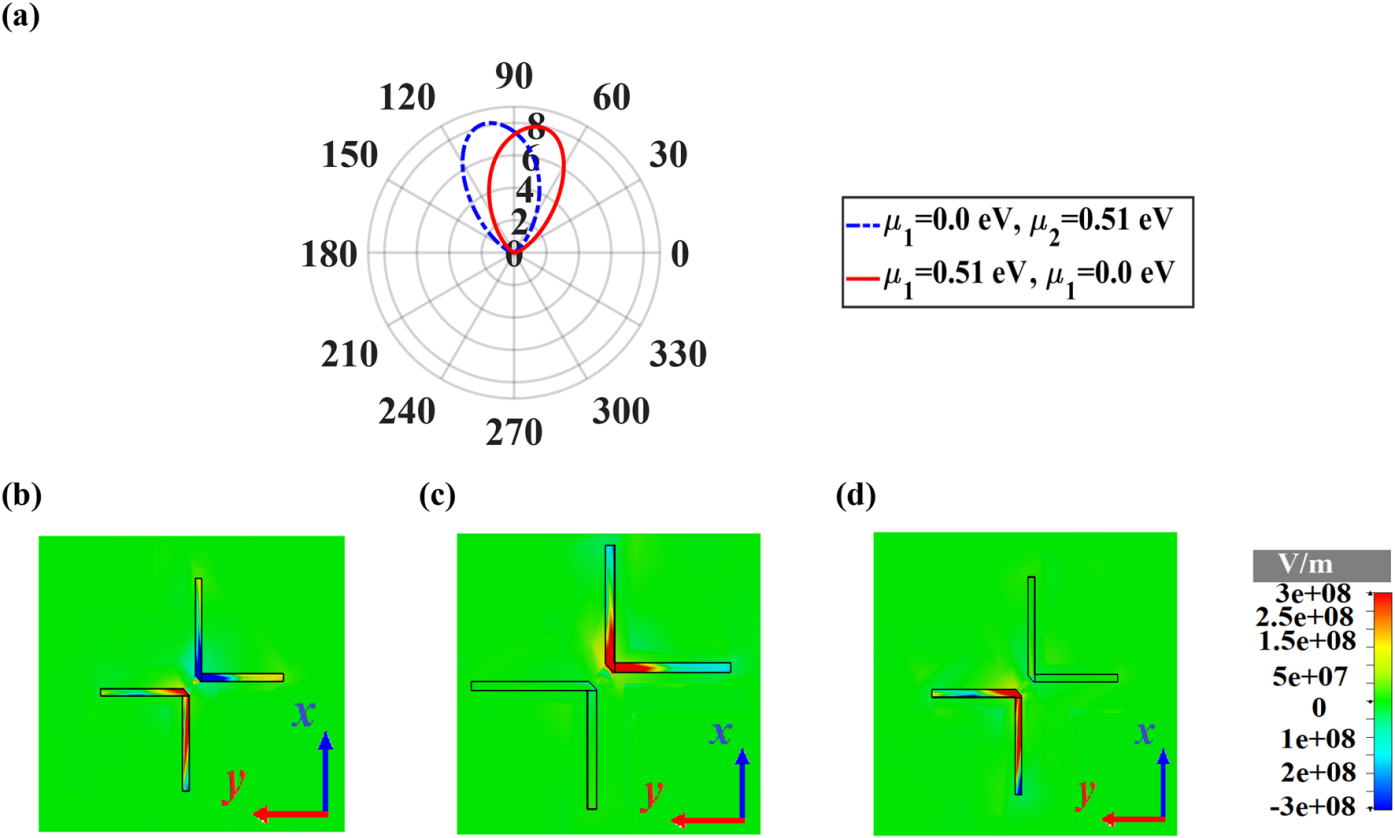
(**a**) 2D polar plot of directivity radiation patterns of smart unidirectional cross dipole nano-antenna at 1550 nm for different states of chemical potential. The distributions of the z-component of electric field at 1550 nm for (**b**) $\mu_{1} = \mu_{2} = 0\,\,{\text{eV}}$, (**c**) $\mu_{1} = 0\,\,{\text{eV}}$, $\mu_{2} = 0\,.51\,{\text{eV}}$ and (**d**) $\mu_{1} = 0\,.51\,{\text{eV}}$, $\mu_{2} = 0\,\,{\text{eV}}$.

Figure 10a depicts that by applying the voltage and so changing the chemical potential of each side, the direction of the main lobe changes, dynamically. When the chemical potential of each side is the same as $\mu_{1} = \mu_{2} = 0\,\,{\text{eV}}$, the graphene acts as a transparent layer. Therefore, the nano-antenna has a unidirectional radiation pattern with the maximum directivity of 8.93 dBi. By choosing different chemical potentials as $\mu_{1} = 0\,\,{\text{eV}}$ and $\mu_{2} = 0.51\,\,{\text{eV}}$ or vice-versa, the unidirectional radiation pattern shifts ± 13° at the wavelength of 1550 nm. The distributions of the z-component of electric field for different states of chemical potential are plotted in Fig. 10b–d. By choosing a suitable chemical potential, the excitation of SPPs and radiation of each arm can be controlled, which leads to tuning the radiation pattern. When $\mu_{1} = 0\,\,{\text{eV}}$ and $\mu_{2} = 0\,\,{\text{eV}}$, the SPPs of both arms are stimulated. In contrast, by choosing different chemical potential for each arm, only the SPPs in one arm with the chemical potential of 0 are excited.

**Figure 11:**
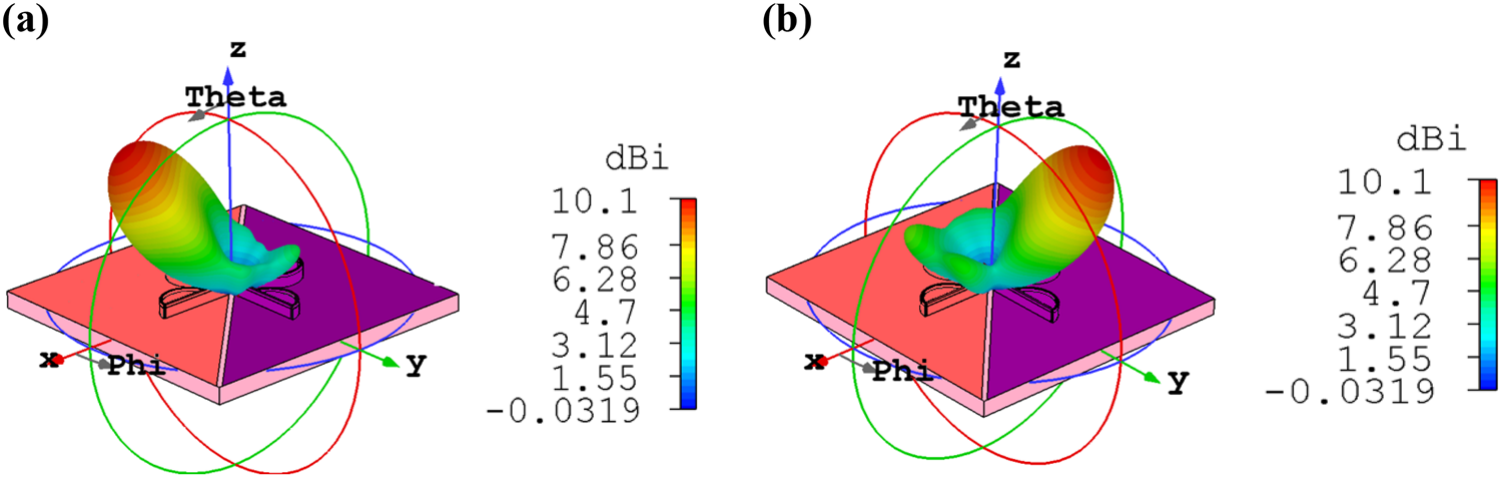
3D radiation patterns of bidirectional cross dipole nano-antenna which acts as the unidirectional one dynamically, when (**a**) $\mu_{1} = 0\,\,{\text{eV}}$, $\mu_{2} = 0\,.51\,{\text{eV}}$ and (**b**) $\mu_{1} = 0\,.51\,{\text{eV}}$, $\mu_{2} = 0\,\,{\text{eV}}$.

In the proposed graphene-based bidirectional nano-antenna, by choosing different values of chemical potential, the bidirectional radiation pattern can modify to unidirectional one. When $\mu_{1} = 0.51\,\,{\text{eV}}$ and $\mu_{2} = 0.51\,\,{\text{eV}}$, graphene has an absorptive performance. Therefore, most radiation electromagnetic lightwave is absorbed by the graphene. As a result, the cross dipole nano-antenna cannot radiate from one side with the chemical potential of 0.51 eV and makes it possible to create a unidirectional pattern with ± 34.6° main lobe direction, as shown in Fig. 11a,b. It is underlying that in comparison to the bidirectional nano-antenna, the unidirectional one has higher directivity of 10.1 dBi, which is due to tuning the radiation pattern in one direction.

**Figure 12:**
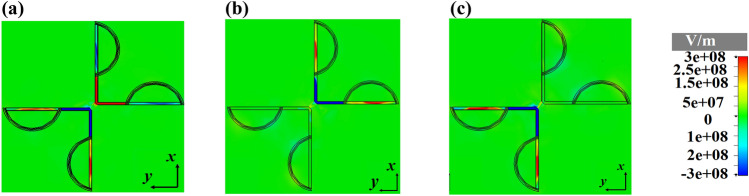
The distributions of the z-component of electric field of smart unidirectional cross dipole nano-antenna at 1550 nm for different states of chemical potential (**a**) $\mu_{1} = \mu_{2} = 0\,\,{\text{eV}}$, (**b**) $\mu_{1} = 0\,\,{\text{eV}}$, $\mu_{2} = 0\,.51\,{\text{eV}}$ and (**c**) $\mu_{1} = 0\,.51\,{\text{eV}}$, $\mu_{2} = 0\,\,{\text{eV}}$.

Based on the obtained results of Fig. 12, it is obvious that the effect of chemical potential of graphene on the excitation and radiation of SPPs for smart bidirectional cross dipole nano-antenna is exactly the same as unidirectional one.

**Figure 13:**
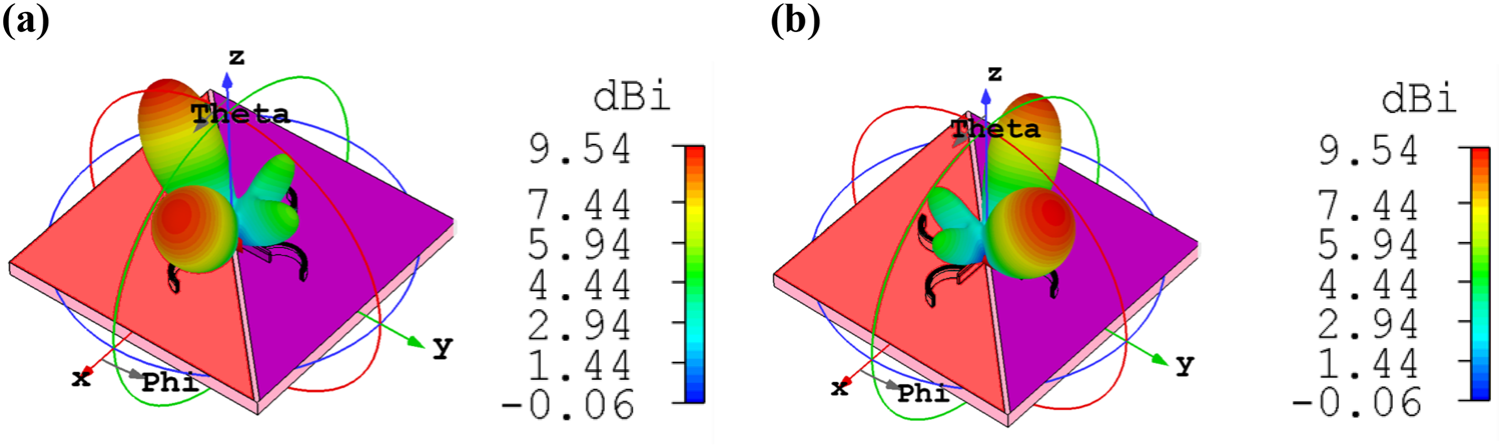
3D radiation patterns of bidirectional cross dipole nano-antenna when (**a**) $\mu_{1} = 0\,\,{\text{eV}}$, $\mu_{2} = 0\,.51\,{\text{eV}}$ and (**b**) $\mu_{1} = 0\,.51\,{\text{eV}}$, $\mu_{2} = 0\,\,{\text{eV}}$.

The quad-directional cross dipole nano-antenna can be used as a wireless link and its accessibility is controlled by choosing an appropriate chemical potential. The nano-antenna has a bidirectional pattern with the directivity of 9.54 dBi, for $\mu_{1} = 0\,\,{\text{eV}}$ and $\mu_{2} = 0.51\,\,{\text{eV}}$ or $\mu_{1} = 0.51\,\,{\text{eV}}$ and $\mu_{2} = 0\,\,{\text{eV}}$, as illustrated in Fig. 13. Two fundamental behaviors of graphene as an absorptive or transparent medium help us to control the radiation patterns of OMBNA. It is obvious that the monolayer graphene cannot completely absorb the power in one direction. Therefore, a back lobe appears. To absorb the power totally, more than one layer should be used, but the fabrication feasibility will be complicated.

**Figure 14:**
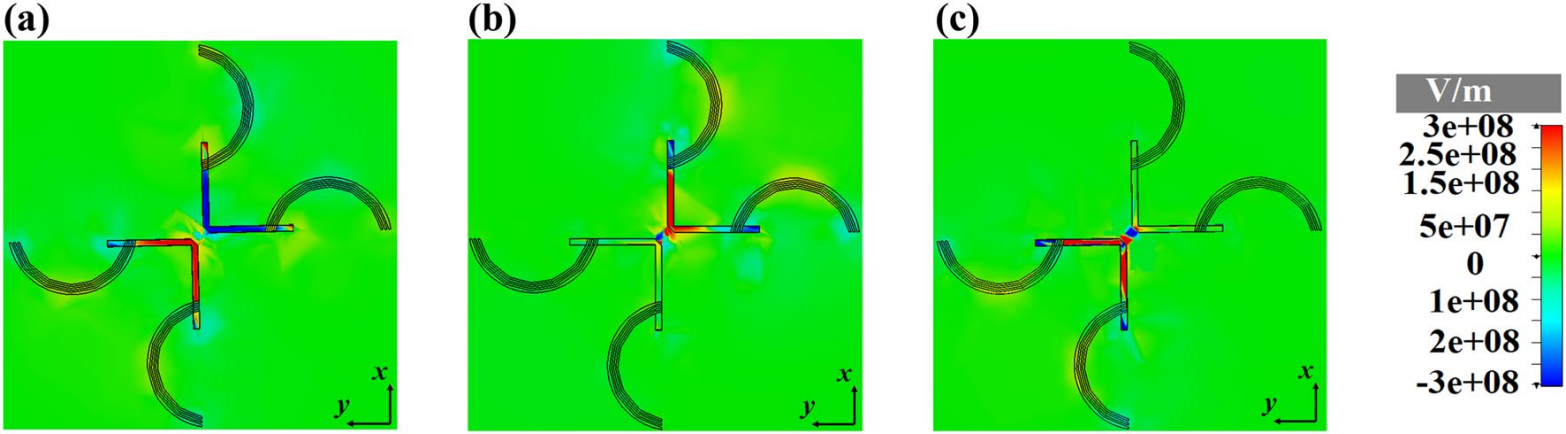
The distributions of the z-component of electric field of smart quad-directional cross dipole nano-antenna at 1550 nm for different states of chemical potential (**a**) $\mu_{1} = \mu_{2} = 0\,\,{\text{eV}}$, (**b**) $\mu_{1} = 0\,\,{\text{eV}}$, $\mu_{2} = 0\,.51\,{\text{eV}}$ and (**c**) $\mu_{1} = 0\,.51\,{\text{eV}}$, $\mu_{2} = 0\,\,{\text{eV}}$.

The z-component of electric field distributions of the smart quad-directional cross dipole nano-antenna are shown in Fig. 14, which confirm the SPPs excitation of the arm with $\mu_{1} = 0\,\,{\text{eV}}$ and $\mu_{2} = 0\,\,{\text{eV}}$.

## Advantages and fabrication feasibility of smart multibeam cross dipole nano-antenna

One of the major features of the proposed smart nano-antenna is modifying the shape of radiation pattern from quad-directional to bidirectional by choosing an appropriate chemical potential of the graphene. Also, in comparison to the spiral antenna^11,14^ with steering angles of 3° and 7°, the cross dipole nano-antenna without the director experiences steering angles of ± 13°by applying enough voltage gate. Moreover, compared to the long dipole nano-antenna to consider higher order resonances for having quad-directional radiation pattern, the length of each arm of the proposed nano-antenna is much shorter than the long rectangular coupled dipole^12^. To design controllable MBNAs, different methods have been used such as SH effect and displacement of the excitation source from the center in spiral antenna. These methods have disadvantages and limitations^11,14,23^ like damaging the structure due to the increase of the input intensity, modifying the radiated wavelength, and complex fabrication process. To overcome these restricts, in this paper, by utilizing the monolayer graphene, we have easily succeeded to suggest the smart OMBNA with different radiation patterns. Finally, to summarize the most important characteristics of our proposed nano-antenna, it has been compared with previous works, as listed in Table 1.

**Table 1:** Characteristics of our proposed smart OMBNA compared to the previous works at 1550 nm.

Reference	Type of radiation pattern	Method of controlling the radiation pattern	Effect of method	Ability of dynamic control	Utilizing graphene	Level of challenge to control the pattern dynamically
^5^	TD	Increasing the flared angle	Modifying the radiation pattern from UD to TD	No	No	–
^11^	UD	Displacement of excitation source from center	Changing the angle of main lobe direction	No	No	–
^12^	BD, QD	Higher order resonances	Modifying the radiation pattern from UD to QD	Yes	No	Medium
^14^	UD	Displacement of excitation source from center	Changing the angle of main lobe direction	No	No	–
^23^	UD	High input intensity	Modifying the radiation pattern from UD to BD and QD	No	No	Hard
Our work	UD, BD, QD	Chemical potential of graphene	Changing the angle of main lobe direction, Modifying the radiation pattern from BD to UD QD to BD	Yes	Yes	Simple

UD unidirectional, BD bidirectional, QD quad-directional, TD triple-directional.

To confirm the fabrication feasibility of the proposed smart cross dipole nano-antenna it should be mentioned that the vertical aspect ratio of this structure is about 20:1, which some methods such as metal assisted chemical etching^40^, nanoimprint lithography and etching^41^ and Talbot lithography^42^ with aspect ratios of (> 420:1), (> 50: 1) and (> 20:1), respectively, can be used to fabricate it. Also, as the gap between each director is 10 nm, the gaps with 4 to 10 nm spacing can be fabricated using a proper e-beam dose and pattern-developing time^43^. Moreover, the scanning electron microscope (SEM) can be used to fabricate gaps less than 2 nm ^44^_._ Consequently, not only the aspect ratio of the proposed smart nano-antenna is not a big challenge, but also the fabrication of sharp gaps between the directors is possible based on the SEM technique. Furthermore, up to now, several experimental studies have been done to fabricate a monolayer graphene, which confirms the fabrication possibility of the suggested smart cross dipole nano-antenna^45–48^.

To concern about the oxidation of silver, it is essential to mention that silver oxidation can be easily prevented by preparing appropriate surfactants such as graphene^49^ and lipid membrane^50^ or setting the nano-antenna in the inert environment such as Argon (Ar) and Helium (He) gases^51^. As the whole proposed nano-antenna is coated by the monolayer graphene, a chemical mechanism happens at the interface of graphene and silver, which is suitable to the reduction of silver oxidation^49^. As a result, graphene plays a major role as a kind of catalyst in deoxidation of silver. Finally, all important features to confirm the fabrication feasibility of the proposed nano-antenna such as aspect ratio, production of single-layer graphene and silver oxidation have been investigated, which prove that in our simulation we have considered all realistic approaches.

## Conclusion

In this paper, for the first time, by utilizing the idea of epsilon-near-zero effect of graphene and designing different arrangements of circular sectors as directors, the smart nano-antenna with bi- and quad-directional patterns has been proposed. Also, we have shown that by introducing the V-shaped cross dipole nano-antenna with the director, its directivity has been enhanced to 12.3 dBi in comparison to the conventional metal cross dipole nano-antenna. Furthermore, by controlling the chemical potential and absorbed power of graphene by electrical gating, the radiation pattern of multibeam nano-antenna can be tuned, dynamically. In this regard, by choosing $\mu = 0.51\,\,{\text{eV}}$, graphene acts as an absorptive layer and hinders the cross dipole nano-antenna to radiate to free space. Therefore, the cross dipole nano-antenna can perform as a switch to control the radiation pattern. By applying an appropriate chemical potential, the bi- and quad-directional nano-antennas can switch to uni- and bi-directional ones. This smart nano-antenna with high directivity is useful for point-to point wireless link and by using graphene the accessibility of users to data can be controlled to enhance the information security.

## Methods

The Lumerical software based on FDTD simulation has been used to obtain the effective refractive index of the proposed nano-antenna by utilizing the mode solution analysis module. The boundary condition of PML with 20 layers has been utilized and the whole structure has been surrounded by air. Also, the experimental Johnson and Christy data has been used to model the silver layer. The far-field performance of the suggested OMBNA has been analyzed based on the full-wave finite element method (FEM) simulations by the CST Microwave Studio software. Moreover, the far field probes have been set far away from the nano-antenna at the wavelength of 1550 nm to obtain the far-field characteristics of the proposed structure, which its resolution is 1°. The graphene has been applied to design a controllable nano-antenna, which its complex surface conductivity has been derived by the Kubo Formula. Furthermore, the adaptive mesh refinements process has been used to enhance the validity of the results. To confirm the accuracy of the achieved results, the nano-antenna is meshed using six segments along both the width and thickness with the 988,989 tetrahedral mesh configuration. Moreover, numerical convergence tests are always performed to ensure the validity of the used mesh.
